# Mitigating Dark Current in Photomultiplication Organic Photodetectors via the Charge Trap Gradient Bulk Heterojunction

**DOI:** 10.1021/acsami.5c11977

**Published:** 2025-08-29

**Authors:** Jing Gao, Zhuangmiao Wang, Yu Tang, Jiayin Han, Mingsheng Gao, Jingnan Wu, Qiaonan Chen, Donghong Yu, Ergang Wang, Furong Zhu

**Affiliations:** † Department of Chemistry and Bioscience, 1004 Aalborg University, 9220 Aalborg, Denmark; ‡ Department of Physics, Research Centre of Excellence for Organic Electronics, Institute of Advanced Materials, 26679 Hong Kong Baptist University, 999077 Hong Kong, China; § Sino-Danish Center for Education and Research, 8000 Aarhus, Denmark; ∥ Department of Chemistry and Chemical Engineering, Chalmers University of Technology, 41296 Göteborg, Sweden

**Keywords:** organic photodetector, photomultiplication, dark current reduction, bulk heterojunction, charge trap gradient

## Abstract

Photomultiplication-type organic photodetectors (PM-OPDs) with dispersed electron or hole traps in a bulk heterojunction (BHJ) have external quantum efficiency far exceeding unity. However, it typically requires a very low donor-to-acceptor ratio, as excess donor or acceptor molecules in the BHJ lead to a high dark current by forming dense charge trap pathways, resulting in hopping conduction. The BHJ layer with a low donor-to-acceptor ratio often associates with a high operating voltage, limiting the use of the PM-OPDs. In this study, we report the results of a new approach to reducing dark current by employing a charge trap gradient design in PM-OPD. This gradient provides two key benefits: (1) it reduces dark current by eliminating charge percolation pathways through regions with low charge trap concentration and (2) it enhances band bending near the electrode by creating regions with high charge trap concentration, facilitating efficient tunneling charge injection. The PM-OPD with a gradient charge trap enables the dark current to be 1 order of magnitude lower than that of an optimal BHJ-based conventional PM-OPD, achieving a high responsivity of 25.40 A/W at 890 nm, operated under 0.3 V, which is nearly 40 times higher than the commercial Si photodiode. These results offer promising opportunities for diverse applications.

## Graphical abstract

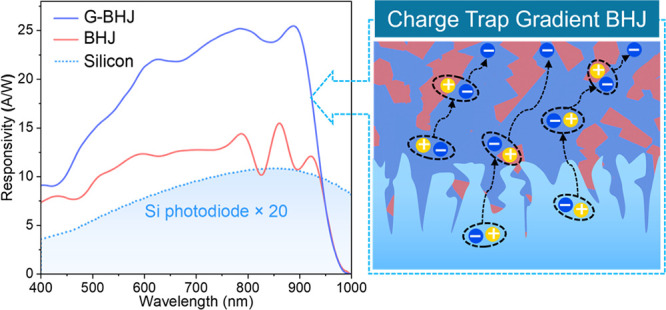

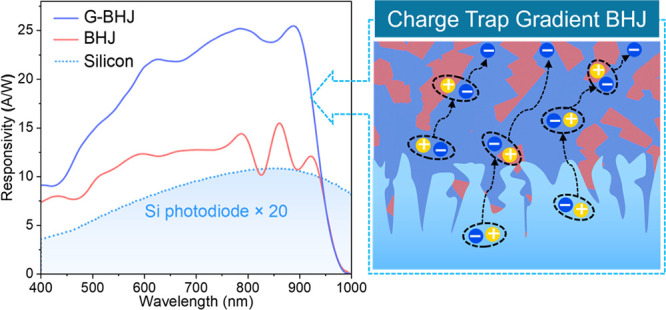

## 1 Introduction

Traditional photodetectors on the market, such as those based on silicon (Si) and III–V compound semiconductors, primarily employ photodiode structures, exhibiting an external quantum efficiency (EQE) typically below 100%.^1–4^ In addition to these inorganic semiconductor-based photodetectors, devices made with organic semiconductors with tunable optoelectronic properties offer additional advantages for large-area flexible photodetectors that can be prepared using low-cost solution fabrication processes.^5–7^ Photodiode-type organic photodetectors (OPDs) are limited to an EQE of 100%. The photomultiplication (PM) effect is demonstrated in OPDs having a homogeneous distribution of charge traps in the bulk heterojunction (BHJ),^8–12^ for example, a binary BHJ with a weight ratio of poly­(3-hexylthiophene-2,5-diyl) (P3HT) to [6,6]-Phenyl C_70_ butyric acid methyl ester (PC_71_BM) of 100:1, achieving an EQE of >100%. In a P3HT:PC_71_BM (100:1)-based PM-OPD, a small fraction of PC_71_BM forms homogeneously dispersed PC_71_BM-induced electron traps in a P3HT:PC_71_BM-based BHJ. Band bending in P3HT occurs due to the accumulation of the trapped photogenerated electrons, caused by the dispersed PC_71_BM-induced electron traps in the BHJ. The band bending leads to tunneling hole injection at the BHJ/electrode interface in a P3HT:PC_71_BM (100:1)-based PM-OPD operated under an operating voltage.^13–15^

Different BHJ PM-OPDs having homogeneously dispersed electron or hole traps in the BHJ have been widely reported.^16–19^ Under illumination, light-induced current can be produced due to trap-assisted charge injection at the BHJ/electrode interface under an operating voltage. However, the ratios of donors to acceptors in the BHJ that produce either electron or hole traps must be very low.^20–23^ An excess fraction of donor molecules creating hole traps or an extra fraction of acceptor molecules producing electron traps in a BHJ results in a high leakage current or a dark current in these PM-OPDs under an operating voltage.^24–26^ This occurs because a high concentration of charge traps in the absorber forms a percolation pathway for charges through hopping conduction in the BHJ under an electrical field.^27,28^ Therefore, existing BHJ PM-OPDs with well-dispersed charge traps in the BHJ, either hole traps or electron traps, require a low donor-to-acceptor ratio. The performance of these BHJ PM-OPDs deteriorates as the donor-to-acceptor ratio shifts away from the respective optimal values. The PM-OPDs, made with well-dispersed electron or hole traps, are often associated with an inherently high operating voltage and a large dark current, resulting in limited sensitivity. Developing a key technical solution to overcome this challenge remains an open issue.

Several approaches have been reported to address the issue, such as incorporating a binary or ternary blend system in BHJ PM-OPDs.^29,30^ However, each approach has its own technical limitations for specific device designs. For instance, utilizing the PM effect is an effective approach to enhance the sensitivity of PM-OPDs in the long wavelength range or under weak light conditions due to trap-assisted tunneling charge injection at the BHJ/electrode interface. In a related work, we developed a bias-controllable dual-band PM-OPD that has a short-wavelength (visible)-light-absorbing layer/spacer/long-wavelength (NIR) light-absorbing layer-based trilayer photomultiplication structure.^31^ An NIR-induced photocurrent is produced by enhanced charge injection via a tunneling effect at the metal interface with the NIR-absorbing layer when operated under a reverse operating voltage. The trilayer PM-OPD responds to short-wavelength (visible) light when operated under a forward operating voltage, enabled by enhanced charge injection at its front contact with the visible-light-absorbing layer. These two operating conditions give the trilayer PM-OPD a dual-band sensing capability for long-wavelength (NIR) light and short-wavelength (visible) light. However, the PM-OPD with homogeneously distributed charge traps in the photoactive region requires an operating bias greater than 20 V due to the use of a thick organic photoactive layer, e.g., >1.0 μm in this case. This presents some technical challenges for applications in portable and flexible devices where high-performance OPDs with low operating voltage are essential, such as healthcare, wearable electronics, imaging, and optical communication.^32–35^

In this work, we resolve the technical hurdles encountered in PM-OPDs by employing a charge trap gradient BHJ, i.e., having a vertically gradually changing ratio of the donor to acceptor in the photoactive layer. The high trap concentration region in the charge trap gradient BHJ helps to enhance band bending in the charge trap gradient photoactive layer in the electrode vicinity, enabling efficient tunneling charge injection due to accumulation of the trapped photogenerated charges. The low trap concentration region in the BHJ mitigates the dark current by eliminating the charge percolation pathway. For example, a hole-trap gradient BHJ (G-BHJ) was prepared using a combination of 3,9-bis­(2-methylene-((3-(1,1-dicyanomethylene)-6,7-difluoro)-indanone))-5,5,11,11-tetrakis­(4-hexylphenyl)-dithieno­[2,3-d:2′,3′-d′]-*s*-indaceno­[1,2-b:5,6-b′]­dithiophene (IT-4F), 2,2′-((2Z,2′Z)-((12,13-bis­(2-ethylhexyl)-3,9-diundecyl-12,13-dihydro­[1,2,5]­thiadiazolo­[3,4-*e*]­thieno­[2″,3″:4′,5′]­thieno­[2′,3′:4,5]­pyrrolo­[3,2g]­thieno [2′,3′: 4,5]­thieno­[3,2-*b*]­indole-2,10diyl)­bis­(methanylylidene))­bis­(5,6-difluoro-3-oxo-2,3-dihydro-1*H*-indene-2,1diylidene))­dimalononitrile (Y6), and poly­[(2,6-(4,8-bis­(5-(2-ethylhexyl-3-fluoro)­thiophen-2-yl)-benzo­[1,2-b:4,5-b′]­dithiophene))-*alt*-(5,5-(1′,3′-di-2-thienyl-5′,7′-bis­(2-ethylhexyl)­benzo­[1′,2′c:4′,5′c′]­dithio-phene-4,8-dione)] (PM6). A low dark current density (*J*_D_) of 1.57 × 10^–4^ mA/cm^2^ was observed for a G-BHJ PM-OPD with a layer configuration of indium tin oxide (ITO)/ZnO (10 nm)/IT-4F/PM6:Y6/Ag, operated under 0.3 V, which is almost an order of magnitude lower than that of an optimal BHJ PM-OPD (2.04 × 10^–3^ mA/cm^2^), made with a weight ratio of PM6 to Y6 of 7:100. The G-BHJ PM-OPD possesses high responsivity, *R*(λ), of 25.40 A/W and a high detectivity D*­(λ) of 1.58 × 10^13^ Jones over the NIR wavelength at 890 nm. The high-performance PM-OPDs demonstrated in this work open an opportunity for a plethora of applications in areas such as high-resolution image sensing, NIR light detection, security monitoring, artificial intelligence, and optical communication.

## 2 Results and Discussion

### 2.1 G-BHJ PM-OPDs

The cross-sectional view of a PM-OPD comprising a layer configuration of the glass/ITO/ZnO/photoactive layer/Ag is shown in Figure 1a. A schematic diagram illustrating the working principles of a BHJ PM-OPD, operated at a forward bias, producing a tunneling electron injection under illumination, enabled by an accumulation of the trapped photogenerated holes at the BHJ/Ag interface, is shown in Figure 1b and that for a G-BHJ PM-OPD, operated at a forward bias, producing an enhanced tunneling electron injection under illumination, enabled by an accumulation of the high density of trapped photogenerated holes at the G-BHJ/Ag interface, is shown in Figure 1c. The use of a charge trap gradient absorber in the PM-OPD has two advantages: (1) it eliminates the charge percolation pathway to suppress the charge hopping conduction and thereby mitigating the leakage current, enabled by the low charge trap concentration region in the absorber and (2) it helps to enhance the band bending in the absorber near the vicinity of the electrode and thereby facilitating an efficient tunneling charge injection due to an accumulation of a high density of the trapped photogenerated charges, caused by the high charge trap concentration region in the absorber. To illustrate the working principle of a G-BHJ PM-OPD, the performance of a control PM-OPD with a typical PM6:Y6-based BHJ having a distribution of homogeneously dispersed hole traps, prepared using a PM6:Y6 blend system with an optimal weight ratio of PM6 to Y6 of 7:100 and a G-BHJ PM-OPD with a charge trap gradient design in the absorber, made with an IT-4F/PM:Y6 (12:100)-based hole-trap gradient, was studied. The IT-4F/PM:Y6-based G-BHJ was prepared using layer-by-layer (LBL) spin-coating deposition sequentially with the IT-4F precursor solution and the PM6:Y6 mixture solution with a higher weight ratio of PM6 to Y6 of 12:100. LBL deposition of IT-4F and PM:Y6 (12:100) produced a gradual increase in the PM6 content in the vertical direction in the active layer. The resulting IT-4F/PM:Y6 (12:100)-based G-BHJ layer has an extremely low ratio of donor (PM6) to acceptor (IT-4F) of (∼0:100) toward the bottom region in the active layer, whereas a relatively higher ratio of donor (PM6) to acceptor (Y6) of 12:100 is formed toward the upper region of the active layer, creating a PM6-induced hole-trap gradient in the photoactive layer. A high concentration of PM6-induced hole traps in the IT-4F/PM:Y6 (12:100)-based G-BHJ PM-OPD enables more efficient tunneling electron injection at the IT-4F/PM:Y6 (12:100)/rear electrode interface as compared to that at the PM6:Y6 (7:100)/rear electrode interface in a PM:Y6 (7:100)-based BHJ PM-OPD. The presence of an extremely low PM6 concentration region in the IT-4F/PM:Y6 (12:100)-based G-BHJ PM-OPD eliminates the charge percolation pathway, suppressing the charge hopping conduction and acting as a hole-blocking layer, thereby mitigating the leakage current. The use of a charge trap gradient absorber enables simultaneously an efficient tunneling charge injection, assisted by the region with an accumulation of a high concentration of the photogenerated charges, induced by the high charge trap concentration in the G-BHJ, and an effective suppression of leakage current through removal of the charge percolation pathway, helped by the region with a low charge trap concentration in the G-BHJ. The molecular structures of the functional materials. e.g., PM6, Y6, and IT-4F, are shown in Figure S1. The schematic diagram of energy levels of the functional materials used in the PM-OPDs, including the highest occupied molecular orbital and lowest unoccupied molecular orbital levels of the organic semiconductors, as well as the work functions of the electrode materials, is shown in Figure S2.

**Figure 1:**
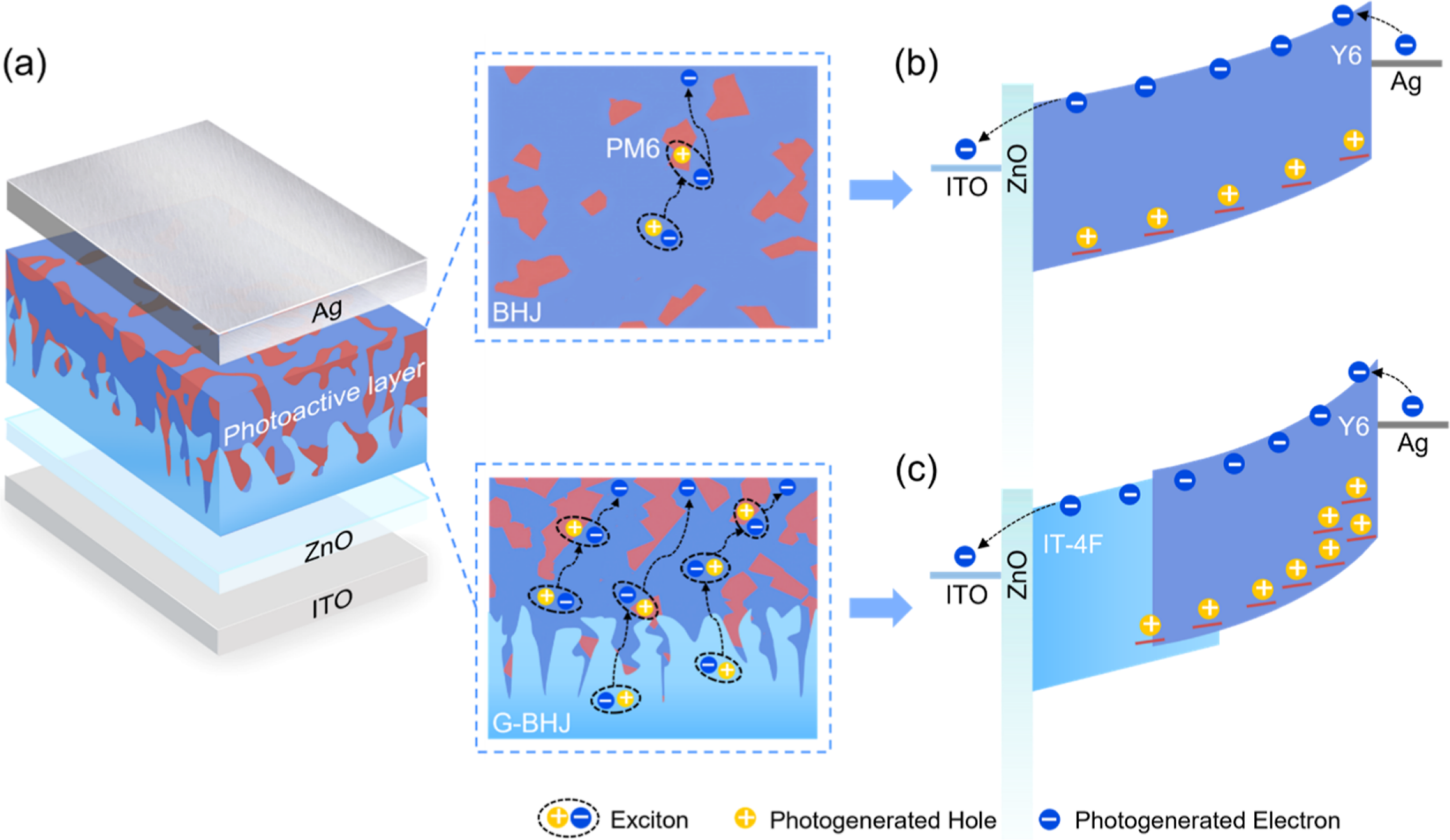
(a) Cross-sectional view of the PM-OPD having a layer configuration of ITO/ZnO/photoactive layer/Ag, prepared using either a BHJ absorber, having an evenly dispersed charge trap, or a G-BHJ photoactive layer having a charge trap gradient in the absorber. Schematic diagrams illustrating the working principles of (b) a BHJ PM-OPD, operated at a forward bias, producing a tunneling electron injection under illumination, enabled by an accumulation of the trapped photogenerated holes at the BHJ/Ag interface, and (c) G-BHJ PM-OPD, operated at a forward bias, producing an enhanced tunneling electron injection under illumination, enabled by an accumulation of the high concentration of trapped photogenerated holes at the G-BHJ/Ag interface.

The miscible PM6 molecules distributed in the PM6:Y6 (7:100)-based BHJ and in the IT-4F/PM:Y6 (12:100)-based G-BHJ act as hole traps in the PM-OPDs. Under a forward bias, a dark current is produced due to the injection of the electrons at the PM6:Y6 (7:100)/rear electrode interface. Under illumination, the tunneling electron injection occurs at the PM6:Y6 (7:100)/rear electrode interface in the BHJ PM-OPD operated at a forward bias due to the band bending that is caused by an accumulation of PM6-induced photogenerated holes, as shown in Figure 1b. In the absence of light, a lower dark current is produced due to the injection of the electrons at the IT-4F/PM:Y6 (12:100)/rear electrode interface in the G-BHJ PM-OPD operated under a forward bias. Under illumination, an enhanced tunneling electron injection occurs at the IT-4F/PM:Y6 (12:100)-based G-BHJ/rear electrode interface in the G-BHJ PM-OPD, operated under a forward bias due to the enhanced band bending that is caused by the high concentration of the trapped photogenerated holes at the IT-4F/PM:Y6 (12:100)-based G-BHJ/rear electrode interface, induced by the high concentration of the PM6-induced hole traps, as shown in Figure 1c.

### 2.2 Spectral Responses

The EQE spectra recorded for an IT-4F/PM6:Y6 (12:100)-based G-BHJ PM-OPD and a PM6:Y6 (7:100)-based BHJ PM-OPD operated at a forward bias of 0.3 V are shown in Figure 2a. The normalized absorption spectra measured for the functional layers of PM6, IT-4F, and Y6 are shown in Figure S3. It shows that the EQE of a G-BHJ PM-OPD is much higher than that of a BHJ PM-OPD over the wavelength range from 400 to 1000 nm. An IT-4F/PM6:Y6 (12:100)-based G-BHJ PM-OPD has a maximum EQE of ∼4400% at 600 nm, which is >69% higher than that of the PM6:Y6 (7:100)-based BHJ PM-OPD (<2500%), enabled by an enhanced tunneling electron injection. The enhanced EQE of the G-BHJ PM-OPD as compared to that of the BHJ PM-OPD over the broadband spectrum is caused by an increased tunneling electron injection due to the enhanced band bending that is caused by an accumulation of a high concentration of PM6-induced photogenerated holes at the IT-4F/PM6:Y6 (12:100)-based G-BHJ/rear electrode interface.

**Figure 2:**
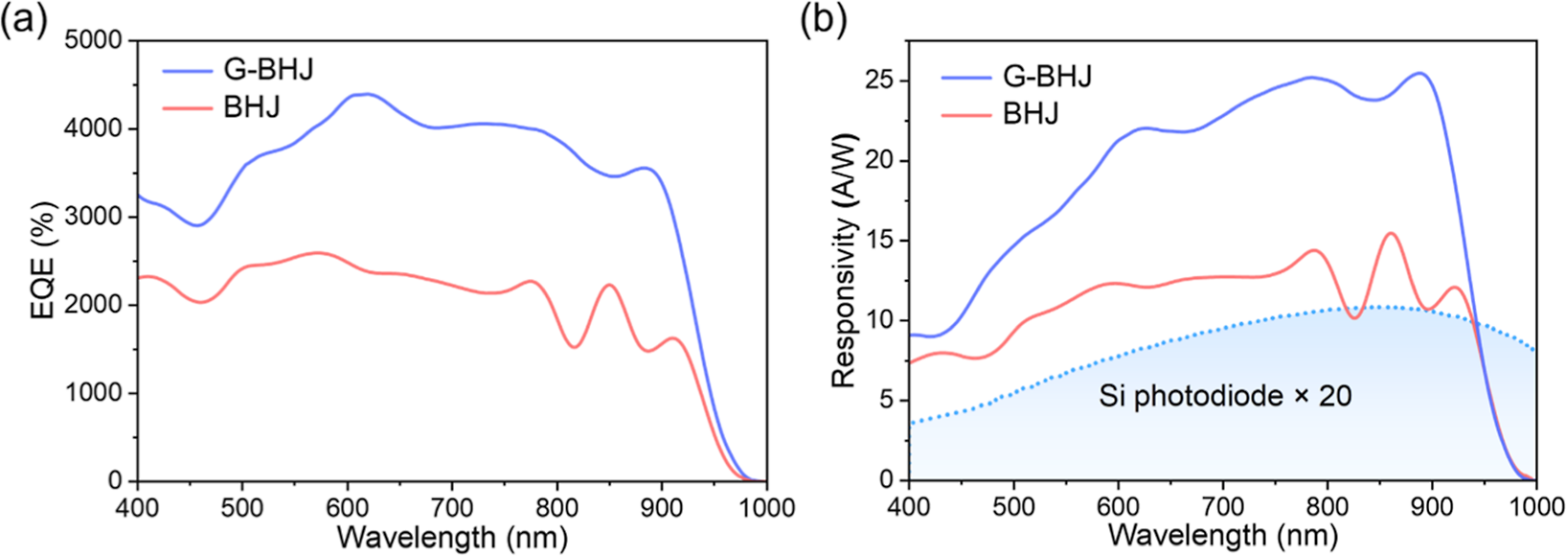
(a) EQE (λ) and (b) *R* (λ) spectra obtained for a G-BHJ PM-OPD and a BHJ PM-OPD operated at 0.3 V. *R* (λ) of a silicon photodiode (enlarged by 20 times) is shown for comparison.

EQE spectra recorded for the BHJ PM-OPDs, operated at a forward bias of 0.3 V and prepared using precursor solutions having different weight ratios of PM6 to Y6 of 5:100, 7:100, 10:100, and 12:100, are shown in Figure S4. The results reveal that a maximum EQE was obtained for a BHJ PM-OPD with an optimal concentration of PM6-induced hole traps in the PM6:Y6-based BHJ, e.g., the absorber prepared using a PM6:Y6 mixture solution having a weight ratio of PM6 to Y6 of 7:100. A decrease in EQE is observed when the BHJ PM-OPDs were prepared using PM6:Y6 precursor solutions having a lower weight ratio of PM6 to Y6 due to a reduced tunneling electron injection. It shows that an increase in the concentration of PM6-induced hole traps in BHJ, e.g., an absorber, prepared using a PM6:Y6 mixture solution with a higher weight ratio of PM6 to Y6 of 12:100, does not help to increase the EQE of the BHJ PM-OPD. Although an increase in tunneling electron injection would occur at the BHJ/rear electrode interface, a high leakage current is also observed for the BHJ PM-OPD having a high concentration of PM6-induced hole traps, caused by the charge hopping conduction via the charge percolation pathway, formed due to a high charge trap concentration in the BHJ. The use of a low charge trap concentration can help to mitigate the leakage current, however limiting the tunneling charge injection. This reveals that the BHJ PM-OPD, e.g., made with a PM6:Y6-based BHJ having a distribution of homogeneously dispersed PM6-induced hole traps, has an inherent limitation to further increase in EQE.

The responsivity, *R*(λ), of the OPDs as a function of the wavelength is calculated using the following equation:

1 $$R\left(\lambda \right)=EQE\left(\lambda \right)\frac{q}{hv}$$

where *q* is the elemental charge and *h*ν is the photon energy. *R*(λ) spectra over the wavelength range from 400 to 1000 nm, obtained for an IT-4F/PM6:Y6 (12:100)-based G-BHJ PM-OPD and a PM6:Y6 (7:100)-based BHJ PM-OPD operated at 0.3 V, are shown in Figure 2b. The *R*(λ) of a Si photodiode (enlarged by 20 times) also is shown for comparison study. It shows that the *R*(λ) of the PM-OPDs is evidently higher than that of the Si photodiode over the wavelength range from 400 to 1000 nm, especially over the NIR wavelength range, demonstrating the advantage of the PM-OPDs for photodetection application. The *R*(λ) of the IT-4F/PM6:Y6 (12:100)-based G-BHJ PM-OPD is significantly higher than that of the optimal PM6:Y6 (7:100)-based BHJ PM-OPD over the broadband wavelength range, with a maximum *R*(λ) of 25.40 A/W at 890 nm, which is more than 66% higher than that of the PM6:Y6 (7:100)-based BHJ PM-OPD (15.29 A/W, at 850 nm). It becomes clear that the G-BHJ PM-OPD has a higher spectral sensitivity as compared to that of the BHJ PM-OPD operated under the same condition. The interference fringes in Figure 2 arise from optical interference within the thin film devices. Both BHJ and G-BHJ PM-OPDs have a 550 nm active layer, but the G-BHJ comprises a bilayer of IT-4F (350 nm) and PM6:Y6 (200 nm), creating an intermixed, less smooth interface between IT-4F and PM6:Y6 layers. This interface increases light scattering and disrupts wave coherence, resulting in less pronounced interference fringes in the G-BHJ compared to the smoother, more uniform BHJ control device. Thus, the G-BHJ PM-OPD exhibits weaker interference effects.

### 2.3 Mitigation of the Dark Current

Current density–voltage (*J*–*V*) characteristics measured for a G-BHJ PM-OPD and a BHJ PM-OPD, operated under a forward bias of 0.3 V, in the dark and under illumination of an 810 nm LED light source with a power density of 4.8 mW/cm^2^ are shown in Figure 3a. Under the same operation condition, a dark current, *J*_D_, of 1.57 × 10^–4^ mA/cm^2^ was observed for a G-BHJ PM-OPD, which is almost an order of magnitude lower than that of an optimal BHJ PM-OPD (2.04 × 10^–3^ mA/cm^2^). The results demonstrate clearly that the presence of a low charge trap concentration region in a G-BHJ PM-OPD provides an effective solution for mitigating the dark current due to the suppression of charge hopping conduction through the charge percolation pathway, which would otherwise occur in a BHJ PM-OPD. Under illumination of an 810 nm LED light source (4.8 mW/cm^2^), a current density, *J*_L_, of 20.5 mA/cm^2^ was obtained for a G-BHJ PM-OPD operated at 0.3 V, which is evidently higher than that observed for an optimal BHJ PM-OPD (1.43 mA/cm^2^). A higher *J*_L_ is realized by an enhanced tunneling electron injection at the G-BHJ/rear electrode interface in a G-BHJ PM-OPD due to an enhanced band bending that is caused by an accumulation of the trapped photogenerated holes at the IT-4F/PM:Y6 (12:100) based G-BHJ/rear electrode interface. *J*–*V* characteristics measured for a G-BHJ PM-OPD under illumination of LED light sources with different peak emission wavelengths of 528, 670, and 810 nm are shown in Figure S5, demonstrating an IT-4F/PM6:Y6 (12:100)-based G-BHJ PM-OPD is very suitable for high-sensitivity NIR detection.

**Figure 3:**
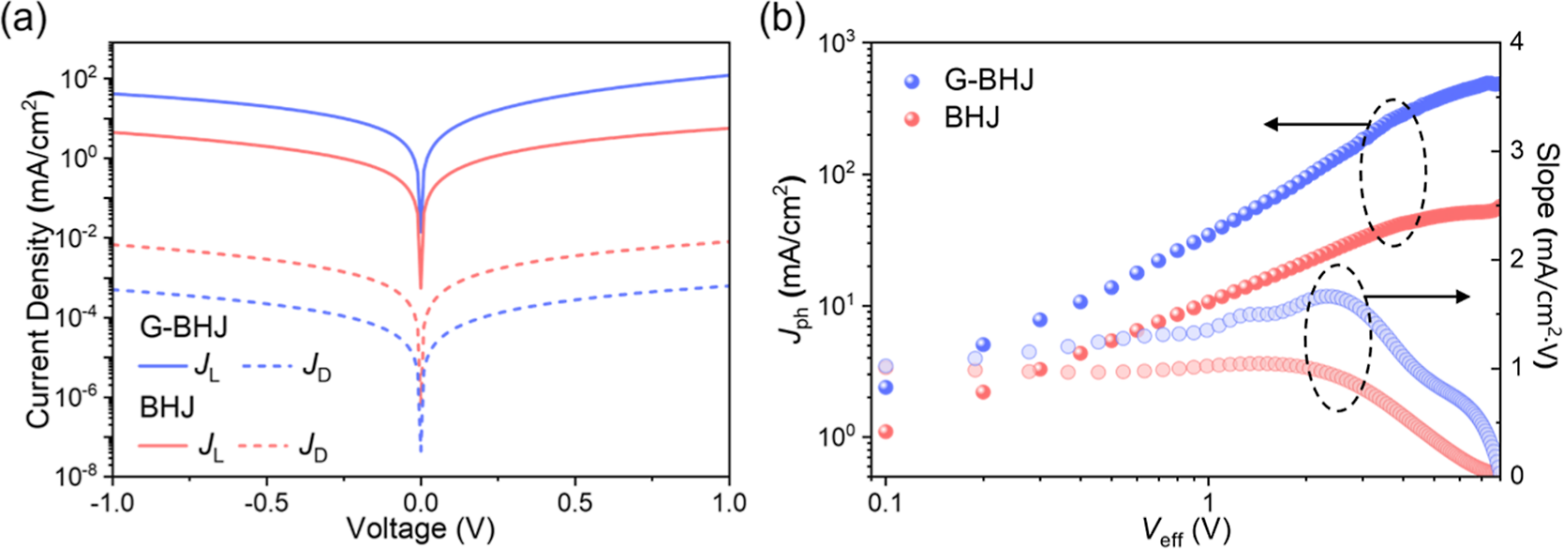
(a) *J*–*V* characteristics measured for a G-BHJ PM-OPD and a BHJ PM-OPD in the dark and under illumination of an 810 nm LED light source with an intensity of 4.8 mW/cm^2^. (b) Slope of *J*_ph_–*V*_eff_ characteristics and *J*_ph_–*V*_eff_ characteristics (in log–log plot) measured for a G-BHJ PM-OPD and a BHJ PM-OPD.

The photocurrent density–effective voltage (*J*_ph_–*V*_eff_) characteristics of the PM-OPDs were analyzed. *J*_ph_ = *J*_L_–*J*_D_, where *J*_L_ and *J*_D_ are the current densities measured for PM-OPDs under illumination and in the dark. *V*_eff_ = *V*_0_–*V*_a_, where *V*_0_ is the built-in potential in the device and *V*_a_ is the applied bias. The charge accumulation behavior in a PM-OPD, PM6-induced hole traps in this case, can be analyzed using the slope of the *J*_ph_–*V*_eff_ characteristics

2 $$Slope=\frac{d\left(\log J_{ph}\right)}{d\left(\log V_{eff}\right)}$$

The slope of *J*_ph_–*V*_eff_ characteristics and *J*_ph_–*V*_eff_ characteristics (in log–log plot) measured for a G-BHJ PM-OPD and a BHJ PM-OPD are shown in Figure 3b. It shows that the G-BHJ PM-OPD has a clearly higher *J*_ph_ as compared to that obtained for the BHJ PM-OPD. There is an apparent hump in the slope of *J*_ph_–*V*_eff_ characteristics measured for the G-BHJ and BHJ PM-OPDs operated at a low voltage, suggesting the presence of an accumulation of the photogenerated holes in the active layers. It becomes clear that the accumulation of the trapped photogenerated holes at the G-BHJ/Ag interface is more prominent as compared to that at the BHJ/Ag interface in an optimal BHJ PM-OPD due to the accumulation of a high concentration of the photo generated holes and thereby facilitating a greater tunneling electron injection as a result of an enhanced band bending in the G-BHJ, as shown in Figure 1b,c.

The photocurrent–light intensity (*J*_ph_–*I*) characteristics measured for an IT-4F/PM6:Y6 (12:100)-based G-BHJ PM-OPD and a PM6:Y6 (7:100)-based BHJ PM-OPD, using an NIR (810 nm) LED light source, operated at a forward bias of 0.3 V are shown in Figure S6. The dynamic range of both types of PM-OPDs was examined using the *J*_ph_–*I* relationship (in log–log plot) over the light intensity range from 10^–6^ to 10 mW/cm^2^. The results reveal that the G-BHJ PM-OPD possesses a higher dynamic range of >90 dB, which is more favorable to that of 70 dB obtained for an optimal BHJ PM-OPD, operated under the same conditions. In addition to the low *J*_D_ and high *J*_ph_, the G-BHJ PM-OPD possesses a broader dynamic range and can detect a weak light level of 2.20 × 10^–5^ mW/cm^2^.

Noise spectral density (*S*_n_) measured for an IT-4F/PM6:Y6 (12:100)-based G-BHJ PM-OPD and a PM6:Y6 (7:100)-based BHJ PM-OPD, operated at a forward bias of 0.3 V, are shown in Figure 4a. *S*_n_ is the fast Fourier transform of the dark current as a function of time. At a frequency of 100 Hz, an *S*_n_ of 5.21 × 10^–13^ AHz^–1/2^ was obtained for an IT-4F/PM6:Y6 (12:100)-based G-BHJ PM-OPD, which is about an order of magnitude lower than 6.30 × 10^–12^ AHz^–1/2^ measured for a PM6:Y6 (7:100)-based BHJ PM-OPD. *S*_n_ results agree with the analyses made with the dark current measurement. It shows that the performance of the G-BHJ PM-OPD is superior to that of the BHJ PM-OPD, having the advantage of low dark current and high photoresponse and thereby achieving a high-sensitivity PM-OPD operated at a low operation voltage.

**Figure 4:**
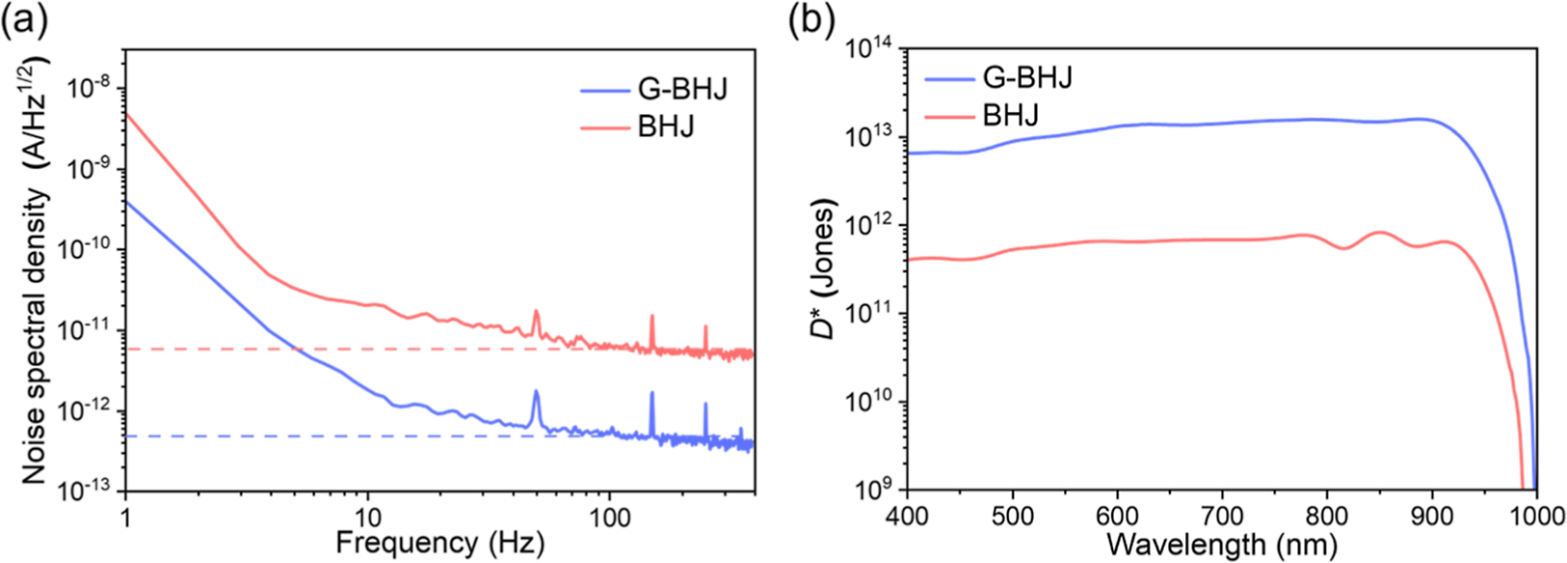
(a) *S*_n_ and (b) D*­(λ) spectra obtained for a G-BHJ PM-OPD and a BHJ PM-OPD operated at 0.3 V.

Specific detectivity, D*­(λ), is an essential characteristic of the photodetector that is associated closely with the noise. It also is one of the main factors describing the ability of detecting NIR electromagnetic waves with a low intensity. D*­(λ), in cm Hz^1/2^/W or Jones, is wavelength dependent, which is related to *R* (λ) and *S*_n_ according to the following equation:

3 $$D^{*}\left(\lambda \right)=\frac{\sqrt{A}\times R\left(\lambda \right)}{S_{n}}$$

where *A* is the active area of the photodetector. D*­(λ) of >5.0 × 10^12^ Jones over the wavelength range from 400 to 1000 nm was obtained for a G-BHJ PM-OPD operated at 0.3 V, which is more than an order of magnitude higher than that obtained for a BHJ PM-OPD, as shown in Figure 4b. The results reveal that an IT-4F/PM6:Y6 (12:100)-based G-BHJ PM-OPD has a high photoresponse with a maximum D* of 1.58 × 10^13^ Jones at 860 nm, which is significantly higher than that of 8.28 × 10^11^ Jones obtained for an optimal PM6:Y6 (7:100)-based BHJ PM-OPD operated under the same conditions. The remarkable improvement in D*­(λ) of a G-BHJ PM-OPD demonstrates its great potential for detecting long wavelength electromagnetic waves with high sensitivity. The spectral response of a G-BHJ PM-OPD is apparently more favorable as compared to that of an optimal BHJ PM-OPD. A comparison of device performance for conventional BHJ PM-OPDs with electron or hole traps reported by different research groups and G-BHJ PM-OPDs developed in this work is listed in Table 1.

**Table 1:** Comparison of Device Performance for Conventional BHJ PM-OPDs with Electron or Hole Traps Reported by Different Research Groups and G-BHJ PM-OPDs Developed in This Work

	charge traps	active layer (weight ratio)	spectral range (nm)	EQE (%)	bias (V)	max D* (Jones)	ref
BHJ	electron	P3HT:PTB7-Th:PC71BM (50:50:1)	350–800	3.8 × 10^4^	–25	3.4 × 10^13^	^14^
		P3HT:PC_61_BM (100:1)	300–700	3.8 × 10^4^	–19	2.6 × 10^13^	^16^
		P3HT:PMBBDT:PY3Se-1V (94:6:3)	350–950	1.1 × 10^5^	–15	2.7 × 10^13^	^20^
		P3HT:O-IDTBR (100:1)	300–800	3.7 × 10^3^	–15	1.0 × 10^13^	^21^
		P3HT:IEICO (100:1)	330–810	1.3 × 10^4^	–25	1.4 × 10^11^	^22^
		PBDB-T:N2200:PS (100:2:0.4)	400–700	4.5 × 10^3^	–12	3.0 × 10^13^	^23^
	hole	P3HT:PC_71_BM (5:100)	300–700	3.9 × 10^3^	5.0	3.2 × 10^12^	^24^
		PBDB-T:PYF-T-O (3:100)	310–900	9.0 × 10^3^	4.0	4.2 × 10^12^	^25^
		P3HT:PC_71_BM:Y6 (3:20:80)	300–1000	1.6 × 10^4^	10	6.6 × 10^9^	^26^
		PM6:Y6 (7:100)	400–1000	2.5 × 10^3^	0.3	8.3 × 10^11^	This work
G-BHJ	hole	IT-4F/PM6:Y6 (12:100)	400–1000	4.4 × 10^3^	0.3	1.6 × 10^13^	This work

Conventional high-performance BHJ PM-OPDs typically require high operating voltages (10–40 V) and exhibit large dark currents, which result from a low donor-to-acceptor ratio in hole-induced PM-OPDs or a low acceptor-to-donor ratio in electron-induced PM-OPDs. These characteristics limit their suitability for easy integration with circuitry in portable and wearable electronics. It shows that the G-BHJ PM-OPDs developed in this work operate efficiently at a much lower voltage of just 0.3 V yet still outperform most conventional BHJ PM-OPDs that require 10–25 V, as shown in Table 1. Response times measured for an IT-4F/PM6:Y6 (12:100)-based G-BHJ PM-OPD and an optimal PM6:Y6 (7:100)-based BHJ PM-OPD, operated at 0.3 V, using a square-wave-modulated 810 nm LED light source with an intensity of 2.0 mW/cm^2^, are shown in Figure S7. The G-BHJ PM-OPD has a rise time (τ_r_) of 0.13 ms and a fall time (τ_f_) of 2.1 ms, which are much smaller than a τ_r_ of 1.3 ms and a τ_f_ of 4.5 ms obtained for a BHJ PM-OPD. Response time of the photodetector reflects the photoresponse speed. The τ_r_ of the PM-OPDs is associated with the tunneling charge injection and the charge transport across the active layer, e.g., in the IT-4F/PM6:Y6 (12:100)-based G-BHJ and PM6:Y6 (7:100)-based BHJ. Incorporation of a G-BHJ, e.g., PM6-induced hole-trap gradient IT-4F/PM6:Y6 (12:100) absorber, can enhance the response speed of the PM-OPD through an enhanced tunneling electron injection at the G-BHJ/rear electrode interface. τ_f_ of the G-BHJ and BHJ PM-OPDs are related to the hole trapping and detrapping processes. The change in response time observed in the G-BHJ PM-OPD and BHJ PM-OPD is closely associated with the difference in the tunneling electron injection, electron transport property, and hole trapping and detrapping processes in the IT-4F/PM6:Y6 (12:100)-based G-BHJ and that in the PM6:Y6 (7:100)-based BHJ. It shows that the G-BHJ PM-OPD has a fast photoresponse speed as compared to that of an optimal BHJ PM-OPD.

The difference in space–charge accumulation behavior between a G-BHJ PM-OPD and a control BHJ PM-OPD was further examined using the capacitance–frequency (*C*–*f*) measurements. *C*–*f* characteristics measured for a G-BHJ PM-OPD and a BHJ PM-OPD, operated under a forward bias of 0.3 V, in the dark and under illumination are shown in Figure 5a. Capacitance and frequency in the *C*–*f* characteristics measured for the devices are related by the following equation:

4 C=J/2πfV

**Figure 5:**
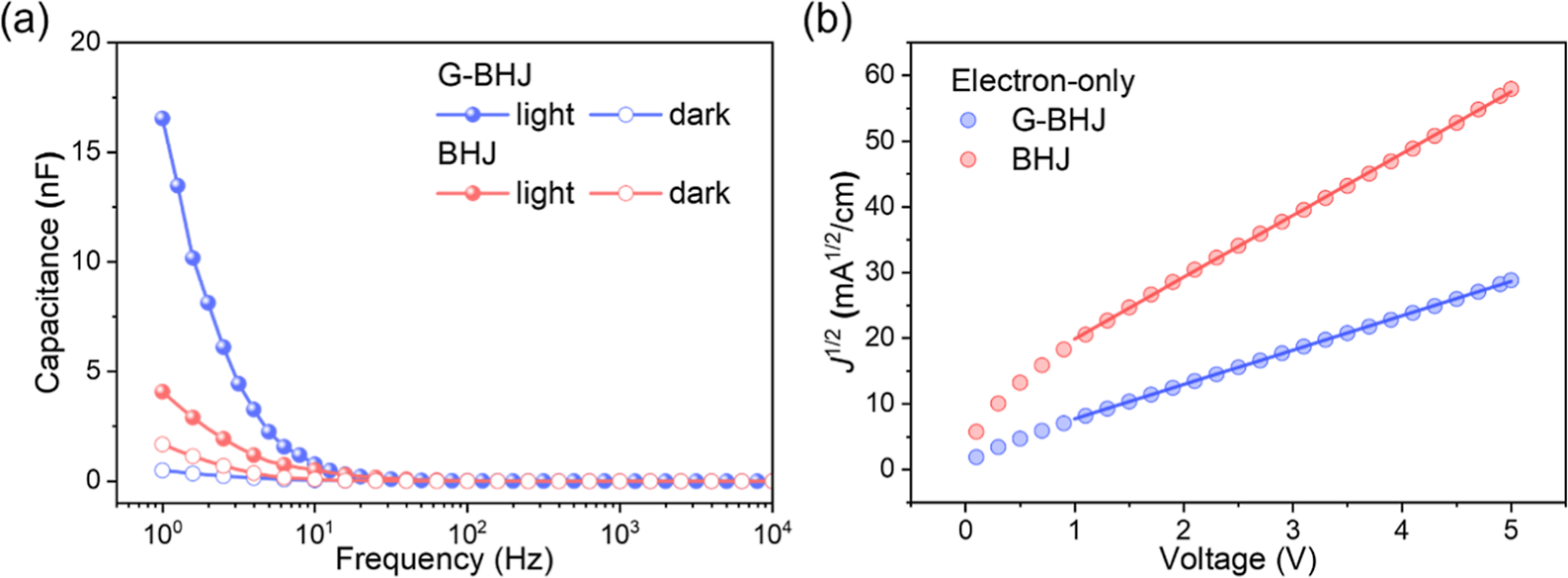
(a) *C*–*f* characteristics and (b) *J* ^1/2^–*V* characteristics obtained for the electron-only devices of ITO/ZnO (60 nm)/G-BHJ (550 nm)/ZnO (60 nm)/Ag and ITO/ZnO (60 nm)/BHJ (550 nm)/ZnO (60 nm)/Ag.

As shown in Figure 5a, both IT-4F/PM6:Y6 (12:100)-based G-BHJ and PM6:Y6 (7:100)-based BHJ PM-OPDs exhibited higher capacitance and current under illumination as compared to that measured for the PM-OPDs in the dark. Under illumination, the *C*–*f* characteristics reveal that the capacitance obtained for an IT-4F/PM:Y6 (12:100)-based G-BHJ PM-OPD is evidently higher than that observed for an optimal PM6:Y6 (7:100)-based BHJ PM-OPD. *C*–*f* measurements suggest that more photogenerated holes are accumulated in the active layer in a G-BHJ PM-OPD, caused by the PM6-induced hole traps, leading to an augmented tunneling electron injection due to the enhanced band bending. *C*–*f* results agree well with the analyses made with the EQE measurements in showing that a high sensitivity PM-OPD can be realized by incorporating a charge trap gradient absorber.

As shown in Figure 5b, the current in the G-BHJ PM-OPD and a BHJ PM-OPD is primary dominated by the electron injection. The charge injection and transport behaviors in both G-BHJ and BHJ PM-OPDs were analyzed using electron-only devices comprising layer configurations of ITO/ZnO (60 nm)/IT-4F/PM6:Y6 (12:100) (550 nm)/ZnO (60 nm)/Ag and ITO/ZnO (60 nm)/PM6:Y6 (7:100) (550 nm)/ZnO (60 nm)/Ag. The electron mobility of the IT-4F/PM6:Y6 (12:100)-based G-BHJ layer and PM6:Y6 (7:100)-based BHJ layer was analyzed using the space–charge-limited current–voltage (SCLC) measurements. Electron mobility, μ_e_, was calculated by fitting the *J*–*V* characteristics measured for the electron-only devices in the dark using the equation

5 $$J=\frac{9}{8}\varepsilon_{0}\varepsilon_{r}\mu \frac{V^{2}}{L^{3}}$$

where μ is the charge mobility, ε_0_ is the permittivity of free space, ε_r_ is the relative permittivity of the active layer, as an approximation ε_r_ is taken for 3 in the calculation, *V* is the voltage, and *d* is the thickness of the active layer. An electron mobility of 1.52 × 10^–3^ cm^2^/(V s) was obtained for a 550 nm-thick IT-4F/PM6:Y6 (12:100)-based G-BHJ layer, which is lower than that of 4.92 × 10^–3^ cm^2^/(V s) obtained for a 550 nm-thick PM6:Y6 (7:100)-based BHJ layer used in an optimal BHJ PM-OPD. As the SCLC measurements were conducted in the dark, there is no accumulation of the trapped photogenerated holes in the active layer, hence the tunneling electron injection is dominated due to the lack of the band bending at the active layer/rear electrode interface. Thus, IT-4F/PM6:Y6 (12:100) (550 nm)- and PM6:Y6 (7:100) (550 nm)-based electron-only devices have similar electron injection behavior. Variation in μ_e_ reflects the difference in the electron transport behaviors in the G-BHJ- and BHJ-based electron devices. A lower μ_e_ of 1.52 × 10^–3^ cm^2^/(V s) obtained for an IT-4F/PM6:Y6 (12:100) (550 nm)-based G-BHJ reveals its relatively poorer electron transport ability as compared to that in a PM6:Y6 (7:100)-based BHJ. A lower μ_e_ observed for an IT-4F/PM6:Y6 (12:100)-based G-BHJ is due to the lack of a charge percolation pathway in its low charge trap concentration portion in the G-BHJ, leading to an effective mitigation of the dark current. Under illumination, the high charge trap concentration region in the G-BHJ allows an increased tunneling electron injection due to an enhanced band bending at the G-BHJ/rear electrode interface, which is desired for achieving a high sensitivity PM-OPD. The analysis made with the SCLC measurements reveals the mechanism of the reduction in dark current in a G-BHJ PM-OPD. A combination of a low dark current and an enhanced tunneling charge injection under illumination is desired for achieving a high-detectivity PM-OPD.

High-performance, low-voltage OPDs with excellent spectral response in the NIR region are crucial for a wide range of applications, including wearable electronics, bioimaging, and optical communication.^32–35^ The results of this work reveal clearly that the use of a charge trap gradient photoactive layer results in a significant increase in spectral response of PM-OPD through a simultaneous reduction in dark current and an increase in tunneling charge injection. The low charge trap concentration region in the charge trap gradient active layer helps to suppress the dark current through mitigating a charge percolation pathway due to the low concentration of charge traps, which would otherwise occur in a conventional PM-OPD with an absorber having an evenly dispersed charge trap. A high charge trap concentration region in the G-BHJ photoactive layer aids in an additional advantage to facilitate the tunneling charge injection due to the accumulation of the trapped photogenerated charges, caused by an enhanced band bending in the photoactive layer near the vicinity of the electrode. A combination of a low dark current and an enhanced tunneling charge injection under illumination is desired for achieving a high-performance PM-OPD.

## 3 Conclusions

In this study, we introduced a novel approach to mitigate the dark current of PM-OPDs using a G-BHJ photoactive layer. This design effectively addresses the challenge of high dark current typically associated with the traditional PM-OPDs, which often require a low donor-to-acceptor ratio and high operating voltage. By creating a charge trap gradient, we successfully reduced the dark current by an order of magnitude as compared to the optimal control PM-OPD and enhanced band bending near the electrode, facilitating efficient tunneling charge injection. The G-BHJ PM-OPD achieved a remarkably high *R* (λ) of 25.40 A/W at 890 nm under a low operating voltage of 0.3 V. This performance is nearly 40 times higher than that of a typical commercial silicon photodiode. The enhanced EQE and D* over a broad wavelength range demonstrate the advantage of the G-BHJ PM-OPDs for applications in high-resolution image sensing, NIR light detection, security monitoring, artificial intelligence, and optical communication.

## 4 Experimental Section

### 4.1 Materials

PM6, Y6, IT-4F, and PEDOT:PSS (PH1000) were purchased from 1-Material.

### 4.2 Device Fabrication

The prepatterned ITO-coated glass substrates with a sheet resistance of ∼15 Ω/square were used as the front electrode for PM-OPDs. ITO/glass substrates were cleaned by ultrasonication sequentially with diluted liquid detergent, deionized water, acetone, and 2-propanol, each for 30 min, and dried by a nitrogen gas flow. The wet-cleaned ITO/glass substrates were exposed to oxygen plasma treatment for 15 min prior to device fabrication. The cleaned ITO glass substrates were then transferred to a nitrogen-purged glovebox with O_2_ and H_2_O levels <0.1 ppm for device fabrication. A 60 nm-thick ZnO electron-transporting layer (ETL) was deposited on the ITO/glass substrate by spin-coating using a ZnO precursor solution at a rotation speed of 3000 rpm for 50 s.

The PM6:Y6-based blend layer was used for making the BHJ PM-OPDs. PM6:Y6 solution having a concentration of 30 mg/mL was formulated by dissolving a PM6:Y6 mixture, with a weight ratio of PM6 to Y6 of 7:100, in a chloroform solvent. A 550 nm-thick PM6:Y6 (7:100) active layer was formed on the ZnO/ITO surface by spin-coating followed by annealing at 150 °C for 60 min. A 550 nm-thick IT-4F/PM6:Y6-based hole-trap gradient active layer was used for the G-BHJ PM-OPDs. The IT-4F/PM:Y6-based G-BHJ was prepared using LBL deposition sequentially with the IT-4F precursor solution and the PM6:Y6 (12:100) mixture solution. IT-4F solution having a concentration of 45 mg/mL was prepared by dissolving IT-4F in chlorobenzene. PM6:Y6 (12:100) solution having a concentration of 30 mg/mL was formulated by dissolving the PM6:Y6 mixture, with a weight ratio of PM6 to Y6 of 12:100, in the chloroform solvent. A 350 nm-thick IT-4F layer was first deposited on top of the ZnO ETL by spin-coating followed by annealing at 150 °C for 30 min. A 200 nm-thick PM6:Y6 (12:100) layer was then overlaid on top of the IT-4F layer by LBL deposition followed by second annealing at 150 °C for 30 min. In a LBL deposition process, the PM6:Y6 (12:100) mixture solution partially dissolves the IT-4F (acceptor) layer, inducing a change in donor (PM6) concentration in vertical direction in the active layer and thereby forming a PM6 gradient with a high PM6 concentration toward the upper surface in the resulting IT-4T/PM6:Y6 (12:100) layer. A 550 nm-thick IT-4F (∼350 nm)/PM6:Y6 (∼200 nm)-based hole-trap gradient G-BHJ sample with a gradual increase in PM6 content toward its upper surface was thus formed by the LBL deposition approach. The PM6:Y6 (7:100)-based BHJ and IT-4T/PM6:Y6 (12:100)-based G-BHJ samples were then transferred to an adjacent vacuum system that connects with the glovebox, with a base pressure of <10^–4^ Pa, for depositing a 100 nm-thick silver (Ag) rear electrode by thermal evaporation. The PM-OPDs have an active area of 3.0 × 3.0 mm^2^, defined by the overlapping area between the front transparent ITO and rear Ag electrodes.

### 4.3 Device Characterization

The monochromatic light source used in the EQE measurement was generated by a xenon lamp and the Bentham TMc300 monochromator. The forward bias used in the transient photoresponse measurements was controlled by the RIGOL DP821A power supply. The *C*–*f* characteristics of the devices were measured in the dark and light, at room temperature, using an impedance analyzer at 0.3 V. The *J*–*V* characteristics of the OPDs were measured using a Keithley-2400 source meter. The hole mobility (μ_h_) and electron mobility (μ_e_) of the IT-4F-, PM6:Y6 (7:100)-, and IT-4F/PM6:Y6 (12:100)-based single-carrier devices were analyzed by using the SCLC technique. The typical *J* ^1/2^–*V* characteristics measured for the electron-only devices of ITO/ZnO (60 nm)/IT-4F/PM6:Y6 (12:100) (550 nm)/ZnO (60 nm)/Ag and ITO/ZnO (60 nm)/PM6:Y6 (7:100) (550 nm)/Zn (60 nm)/Ag are shown in Figure 5b. The analysis made with the charge transport mobility measurements provides an inside look at improving the understanding of the mechanism of the dark current mitigation in the G-BHJ PM-OPDs. The dynamic range of the PM-OPDs was measured under illumination of an NIR (810 nm) light source. The intensity of the incident light was adjusted by using different neutral density filters.

## Supplementary Material



## Notes

Molecular structures and normalized absorption spectra of the functional materials; EQE spectra of the BHJ PM-OPDs prepared using different weight ratios of PM6 to Y6; *J*–*V* characteristics of the G-BHJ PM-OPD under illumination of LED light sources with different peak wavelengths; and *J*_ph_–*I* characteristics and response time of the G-BHJ PM-OPD and BHJ PM-OPD (PDF)
