# Supplementary material for: Mitigating Dark Current in Photomultiplication Organic Photodetectors via the Charge Trap Gradient Bulk Heterojunction
Source: ACS Appl Mater Interfaces. 2025 Aug 29;17(37):52426–34. doi: 10.1021/acsami.5c11977 (PMC12447394; doi:10.1021/acsami.5c11977)
Supplement: Supplementary file 1 [file am5c11977_si_001.pdf]

Supporting Information

Mitigating Dark Current in Photomultiplication Organic Photodetectors via Charge Trap Gradient Bulk Heterojunction

Jing Gao[1,2], Zhuangmiao Wang[2], Yu Tang[2], Jiayin Han[2], Mingsheng Gao[2], Jingnan Wu[1], Qiaonan Chen[3], Donghong Yu[1,3]*, Ergang Wang[4]*, Furong Zhu[2]*

1. Department of Chemistry and Bioscience, Aalborg University, 9220 Aalborg, Denmark.

2. Department of Physics, Research Centre of Excellence for Organic Electronics, Institute of Advanced Materials, Hong Kong Baptist University, 999077 Hong Kong, China.

3. Sino-Danish Center for Education and Research, 8000, Aarhus, Denmark.

4. Department of Chemistry and Chemical Engineering, Chalmers University of Technology, 41296 Göteborg, Sweden.

Emails: yu@bio.aau.dk, ergang@chalmers.se, frzhu@hkbu.edu.hk

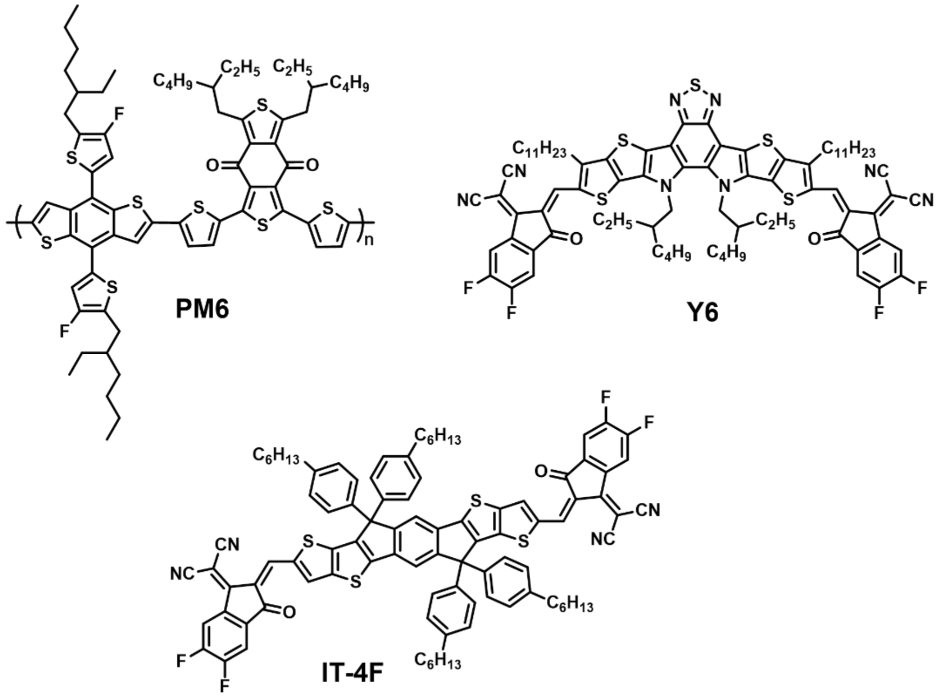

Figure S1. Molecular structures of example functional materials, PM6, Y6 and IT-4F, used in the photodetector

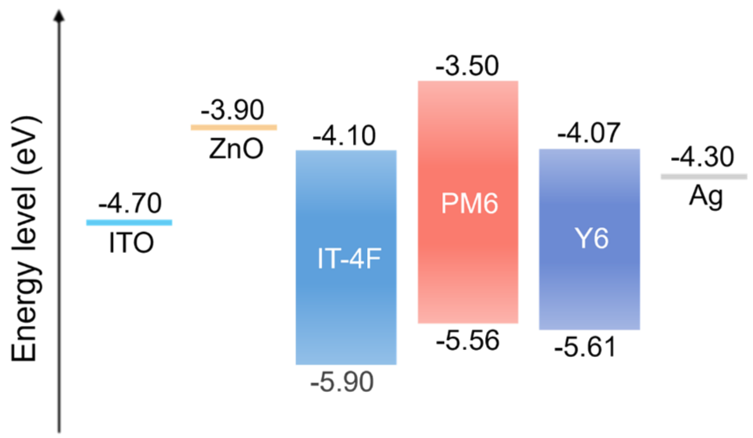

Figure S2. Schematic diagram showing the energy levels of the functional materials used in the photodetector.

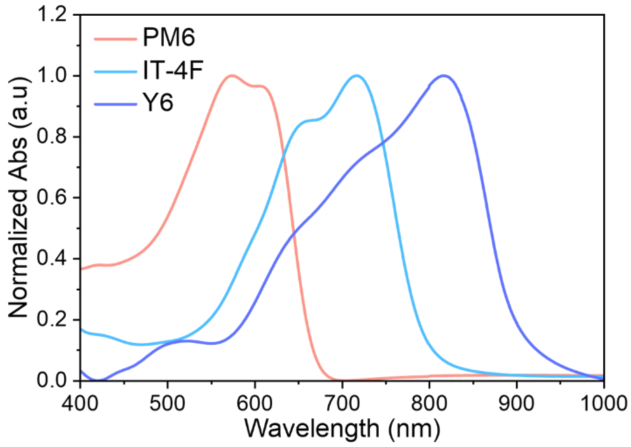

Figure S3. Normalized absorption spectra measured for the thin films of PM6, IT-4F and Y6 functional materials.

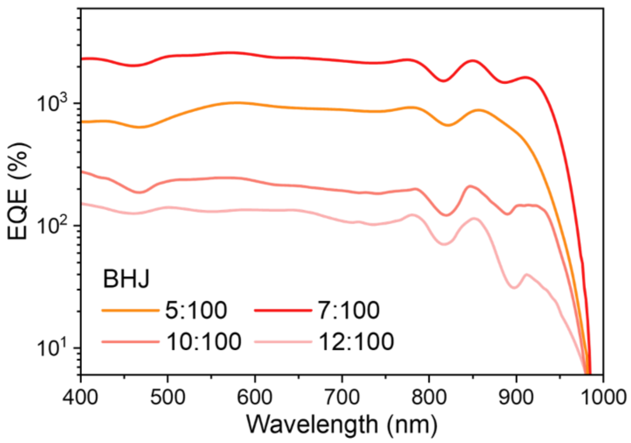

Figure S4. EQE spectra measured for the BHJ PM-OPDs prepared using precursor solutions having different weight ratios of PM6 to Y6 of 5:100, 7:100, 10:100, and 12:100.

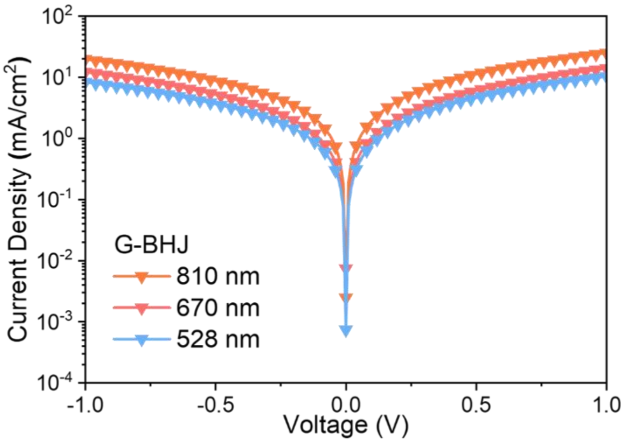

Figure S5. J–V characteristics measured for the G-BHJ PM-OPD under illumination of LED light sources with different peak emission wavelengths of 528, 670 and 810 nm.

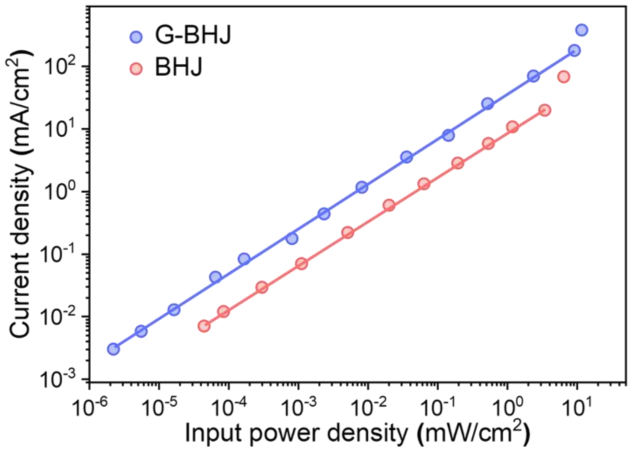

Figure S6. Photocurrent–light intensity (J_{ph}–I) characteristics measured for the G-BHJ PM-OPD and BHJ PM-OPD operated at a forward bias of 0.3 V.

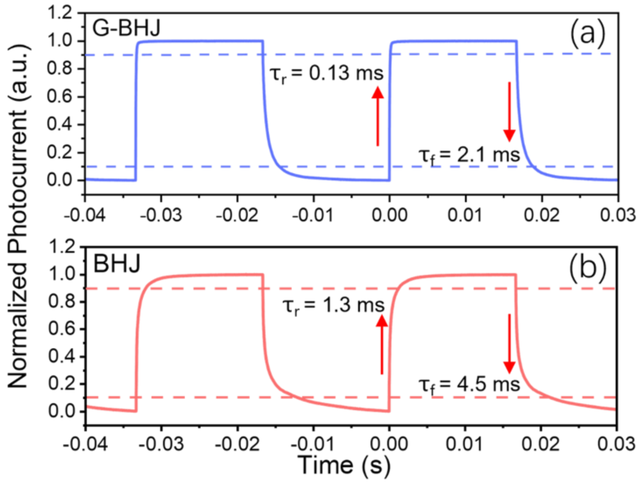

Figure S7. Response time measured for (a) a G-BHJ PM-OPD and (b) a BHJ PM-OPD, operated at 0.3 V, using an 810 nm LED light source with a light intensity of 4.8 mW/cm^2.
